# Multi-Source Porosity Image Normalization (NMI) in Selective Laser Melting for Reliable Reuse of Heterogeneous Microstructural Data

**DOI:** 10.3390/ma18245579

**Published:** 2025-12-12

**Authors:** Shupeng Guo, Xiaoxun Zhang, Fang Ma, Anyong Lu, Yuanyou Huang

**Affiliations:** 1 School of Materials Science and Engineering, Shanghai University of Engineering Science, Shanghai 201620, China; 2 School of Mechanical and Automotive Engineering, Shanghai University of Engineering Science, Shanghai 201620, China

**Keywords:** spatial scale normalization, scale bar detection, porosity image standardization, heterogeneous microstructural data, selective laser melting

## Abstract

Selective laser melting (SLM) is a key technology in metal additive manufacturing (AM), but the widespread presence of porosity defects in fabricated parts significantly degrades mechanical performance and limits practical applications. Machine learning (ML) and deep learning (DL) have shown great potential in porosity prediction, defect detection, and performance modeling. However, their application remains constrained by the lack of systematic “processes–images–properties” datasets and the high cost of experimental data acquisition. To address this challenge, this study proposes an innovative normalization method for multi-source SLM porosity images (NMI). The method integrates scale bar detection and removal, physical size normalization, and resolution harmonization to ensure dimensional consistency while preserving critical pore features. Systematic validation using both literature-derived and experimental datasets demonstrates that NMI effectively integrates heterogeneous image data, enhances dataset consistency, and promotes the reuse of existing imaging resources. This framework provides a scalable and resource-efficient pathway for DL-based defect prediction and process optimization, and establishes a solid foundation for constructing standardized and extensible materials datasets.

## 1. Introduction

Additive manufacturing (AM) has emerged as a transformative manufacturing technology, enabling the cost-effective fabrication of complex three-dimensional structures through layer-by-layer material deposition. Among metal AM techniques, Selective Laser Melting (SLM) is widely recognized for producing near fully dense components with intricate geometries and adapted properties by controlling process parameters such as laser power, scanning speed, layer thickness, and hatch spacing [1,2].

However, improper process parameter settings can lead to defects such as porosity in the fabricated parts. The presence of such defects significantly degrades mechanical strength, fatigue life, and corrosion resistance. Consequently, predicting porosity and detecting defects under varying process parameter conditions has become a critical research objective [3,4,5,6].

Over the past decade, machine learning (ML) and deep learning (DL) have been widely applied to SLM [7,8], encompassing tasks such as pore detection [9], porosity prediction [10], microstructural image generation [11], and mechanical property prediction [12]. The successful application of ML models for porosity prediction largely depends on the availability of a large number of annotated training images [13]. In most existing studies, these images and property data have been obtained through in-house experiments. For instance, Gobert et al. [14] employed optical sensors during powder bed fusion additive manufacturing to capture multiple in situ images under eight different lighting conditions, thereby constructing a supervised ML dataset for defect detection. Imani et al. [15] performed quantitative porosity analysis using X-ray CT images of samples fabricated under varying process parameters, including laser power, scanning speed, and hatch spacing. Aminzadeh and Kurfess [16] compiled a dataset of 35 samples produced with different combinations of laser power and scanning speed, which was then used to train a Bayesian classifier for pore classification. Similarly, Gu et al. [17] fabricated 21 sets of samples with process variables including laser power, scanning speed, hatch spacing, and layer thickness to train DL models for automated segmentation and detection of melt pools and porosity in AM microstructural images. However, acquiring such experimental datasets is not only time-consuming and costly but also constrained by limited parameter coverage and excessive material consumption.

In contrast, published literature contains a vast amount of image and performance data associated with various process parameter combinations and porosity levels. Leveraging these datasets for ML model training, validation, or data analysis can significantly reduce the time and cost required for experimental data acquisition. Several researchers have demonstrated the feasibility of this approach. For instance, Liu et al. [18] trained a rapid melt pool geometry prediction model using experimental data previously reported by Gunenthiram et al. [19], achieving prediction results consistent with both experimental and simulation outcomes. Li et al. [20] collected 276 porosity defect images from multiple publications, encompassing most porosity morphologies, to train a porosity defect detection model. Joy et al. [21] employed Gaussian Mixture Models (GMMs) to generate synthetic data based on collected datasets while preserving their statistical integrity, and subsequently used the expanded dataset to train a deep neural network (DNN) regression model for mechanical property prediction. Notably, although these studies recognized the potential of published literature data for ML model training, the datasets they employed were either derived from a single source or did not address inconsistencies in scale across multi-source images. As a result, they did not fully explore the relationship between process parameters and pore size.

The integration of heterogeneous image data is inherently challenging due to these inconsistencies. Without normalization, variations in pixel dimensions, image formats, and annotation conventions can lead to biased analyses or inconsistent machine learning outcomes. Therefore, this issue has attracted considerable attention in the research community, leading to a growing body of work focused on standardization and harmonization methods for image-based analysis. Hernandez et al. [22] proposed a data framework adhering to the FAIR principles (Findable, Accessible, Interoperable, and Reusable), which addresses the challenge of multimodal data registration in laser powder bed fusion (L-PBF) additive manufacturing. Their approach significantly improves data organization, usability, and compatibility with intelligent analysis workflows. Kim et al. [23] developed a universal and scalable image analysis platform that integrates computer vision and machine learning techniques to automatically extract particle size and morphology from SEM images, providing a more accessible pathway for constructing nanoparticle information datasets. Building upon this, Tao et al. [13] proposed an improved U-Net model enhanced with attention mechanisms and residual networks, which, when combined with Kim et al.’s particle measurement pipeline, achieved fully automated nanoparticle size measurement from SEM images, reaching a mean relative error of just 4.25% compared to manual annotations.

However, research on integrating multi-source image data in the field of selective laser melting (SLM) remains limited. Most existing standardization efforts focus exclusively on individual experimental datasets, neglecting the valuable image data available from other published studies. This narrow approach constrains dataset diversity and significantly impedes the effective application of DL in this domain.

To address these challenges, this study focuses on two core scientific questions: (1) how to develop a spatial scale normalization method that unifies image dimensions while preserving porosity characteristics, and (2) how to construct a systematic dataset linking SLM process parameters, image-based features, and actual performance metrics, thereby establishing a solid foundation for data-driven model development. Currently, the field lacks a standardization framework capable of handling multi-source SLM images.

To this end, this study proposes an image standardization workflow termed Normalization for Multi-source SLM Porosity Images (NMI) based on spatial scale normalization. This method enables consistent physical-scale processing of heterogeneous SLM porosity images by performing scale bar detection and removal, followed by normalization of physical dimensions and image resolution. Specifically, the process begins with annotation masking and binarization to eliminate variations in background across different images. Scale bar information is then accurately extracted and removed to determine the pixel-to-physical size ratio, while ensuring that black scale bars do not interfere with pore structure recognition. Based on the extracted scaling factor, a unified target cropping area in physical dimensions is defined, and each image is subsequently cropped and resampled to a uniform resolution.

We validated the effectiveness of the proposed NMI method using three pore image datasets from distinct sources. On this basis, a comprehensive processes–images–properties dataset was constructed, integrating both literature-derived and experimentally acquired images. To validate the accuracy of the proposed scale bar detection method, its results were quantitatively compared against ground truth measurements as well as values obtained using the ImageJ 1.54f (National Institutes of Health, Bethesda, MD, USA) and the image analysis platform developed by Kim et al. [13,23]. To evaluate normalization performance, metrics such as relative porosity error and deformation coefficient before and after cropping were compared against a conventional single-source cropping approach.

This strategy not only facilitates the efficient reuse of existing experimental image data, addressing the persistent issue of data silos in the additive manufacturing field, but also provides a scalable and resource-efficient foundation for DL-based porosity modeling and SLM process optimization. Furthermore, it demonstrates the practical value and predictive potential of literature-derived images in data-driven workflows.

Based on the aforementioned research motivations and key challenges, the primary objectives of this study are:

1. To propose a multi-scale image normalization method that unifies the spatial scale of multi-source SLM porosity images, addressing inconsistencies in resolution and physical dimensions across datasets;
2. To develop a reliable workflow for scale bar detection and removal, enabling precise extraction of pixel-to-physical dimension ratios and eliminating non-structural, manually embedded annotations from images;
3. To establish a comprehensive processes–images–properties dataset collection platform that effectively integrates the normalized porosity images with corresponding SLM process parameters and material performance indicators, providing a solid foundation for subsequent data-driven modeling.

The remainder of this paper is organized as follows: Section 2 presents the proposed image normalization methodology in detail; Section 3 describes the sources and construction of the three pore image datasets; Section 4 provides the experimental results along with an in-depth discussion; Finally, Section 5 concludes the paper by summarizing the key findings.

## 2. Methodology

**Figure 1:**
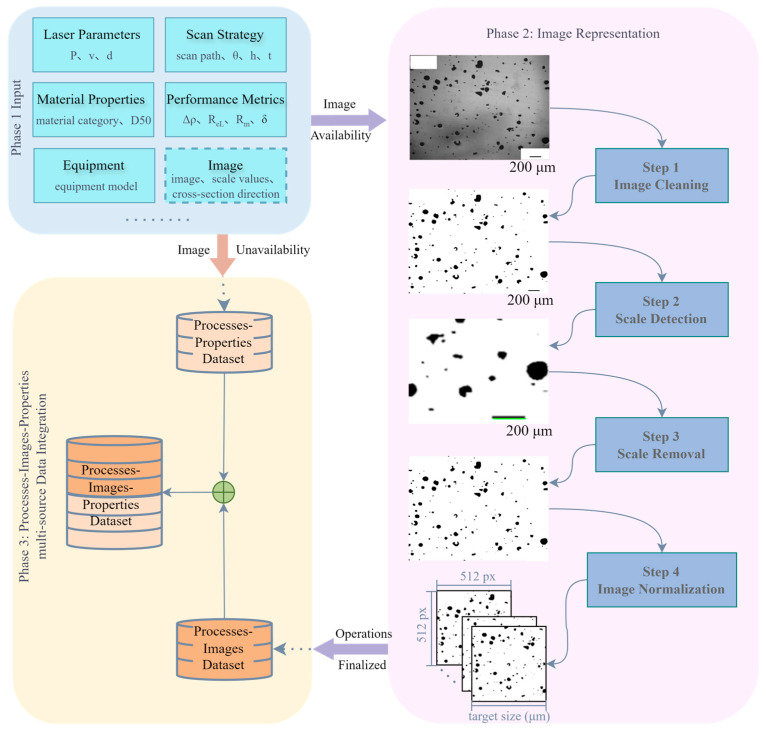
Overall methodology of the proposed SLM processes–images–properties multi-source data integration method.

**Figure 2:**
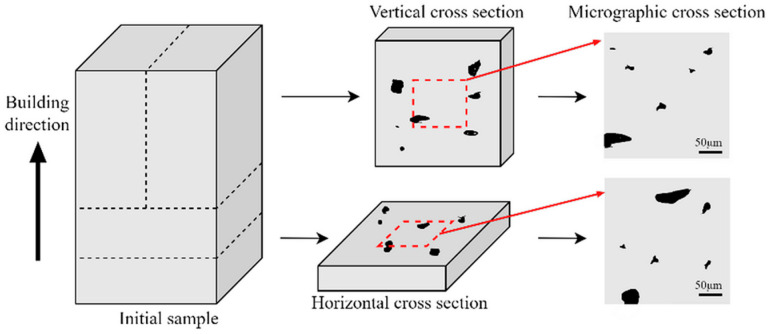
Micrographic cross-sections illustrating typical pore structures in SLM-fabricated 316L stainless steel. Both vertical and horizontal cross-sectional views are shown, each embedded with scale bars and section identifiers. These annotations facilitate accurate interpretation of pore morphology and enable consistent pixel-to-physical dimension calibration in subsequent image analysis workflows.

Figure 1 illustrates the overall framework of the proposed methodology, which involves three phases of SLM processes–images–properties multi-source data integration method. Phase 1 (Input) aims to procure a comprehensive dataset comprising process parameters, equipment model, and corresponding performance metrics alongside micrographic images, sourced from public databases, published literature, and custom experimental trials to complete initial data acquisition. In this phase, each sourced dataset should encompass comprehensive process parameters covering critical SLM configurations, including but not limited to laser characteristics, scan strategies, and material properties, while microstructural characterization images must incorporate scale bars and cross-section identifiers (e.g., vertical/horizontal cross-sections as illustrated in Figure 2) [24]. Corresponding mechanical performance metrics per parameter set shall include essential properties: yield strength (YS), ultimate tensile strength (UTS), elongation at break (EL), or microhardness (HV).

Phase 2 focuses on normalizing multi-source microstructural characterization images. To this end, we propose a method named Normalization for Multi-source SLM Porosity Images (NMI), which systematically aligns the spatial scale and resolution of porosity images acquired from different sources. This approach ensures that all images are expressed in consistent physical dimensions, enabling reliable comparative analysis and subsequent data. The NMI workflow comprises four sequential steps:

(i) Image Cleaning: Microstructural images with disparate backgrounds are converted into binary form, where white pixels correspond to the matrix region and black pixels represent pores. This preprocessing stage promotes accurate scale bar detection in the following step.
(ii) Scale Detection: The pixel width of the scale bar in each image is measured and combined with the given scale factor to establish the pixel-to-physical size ratio, providing a quantitative basis for cross-image scale unification.
(iii) Scale Removal: The scale bar is removed from the binary image to prevent interference in pore structure analysis, yielding images that contain only the porosity features.
(iv) Image Normalization: As the central step of NMI, all binary images from different sources and magnifications are cropped and rescaled to a uniform target physical dimension.

In Phase 3, the dataset that links process parameters to material properties, known as the processes–properties dataset and obtained in Phase 1, is integrated with the processes–images dataset that is generated from standardized images in Phase 2. This integration results in a unified processes–images–properties dataset, which simultaneously contains information on processing conditions, corresponding image representations, and their resulting material properties.

It should be noted that the proposed NMI framework is fundamentally different in purpose from conventional image normalization and harmonization techniques, such as histogram equalization [25], Z-score normalization [26], or medical image harmonization [27]. These existing approaches primarily address intensity or contrast discrepancies across datasets, aiming to ensure visual or statistical consistency. In contrast, NMI is specifically designed to correct physical-scale heterogeneity among multi-source SLM porosity images, where variations in magnification, scale-bar information, and pixel-to-length ratios result in dimensional inconsistency. Therefore, NMI should be considered as a physical dimension standardization approach that complements traditional normalization and harmonization methods, rather than directly competing with them. Its primary contribution lies in enabling the integration of heterogeneous image datasets from different experimental and literature sources for machine learning–based SLM analysis.

### 2.1. Image Preprocessing

Image preprocessing consists of three primary steps: annotation masking, adaptive binarization, and dynamic threshold adjustment. These steps are designed to standardize heterogeneous multi-source images into binarized representations with a uniform background and clearly distinguishable black scale bars.

**Figure 3:**
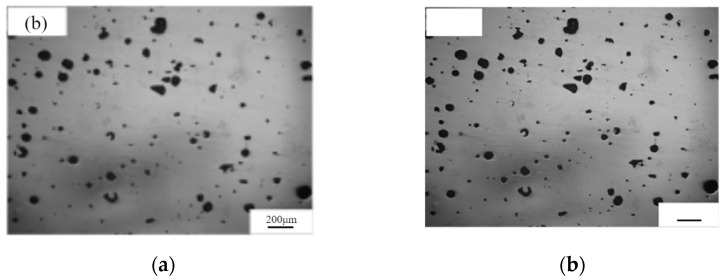
Implementation results of annotation masking: (**a**) Literature-derived original image. (**b**) Processed image after masking extraneous annotations and text information of scale bar, which serves as input for subsequent NMI procedures. Annotation masking enables the removal of noise elements (e.g., the “b” label and “200 µm” text in (**a**)) while preserving pore structures, thereby preventing interference in the following normalization process.

Annotation masking, formally standardized under the ASTM E2809-22 methodology [28], removes extraneous graphical annotations from micrographs by generating location-specific binary masks that isolate and suppress annotations while preserving the native texture and pore information of the image. This approach effectively mitigates the interference caused by annotation elements commonly found in literature-sourced images. Specifically, the masking process overlays white binary masks on annotated regions, preventing any alteration to the underlying pore structures. Consequently, annotation masking eliminates non-structural noise prior to binarization, ensuring the integrity of pore features during subsequent segmentation steps. The implementation results are illustrated in Figure 3.

**Figure 4:**
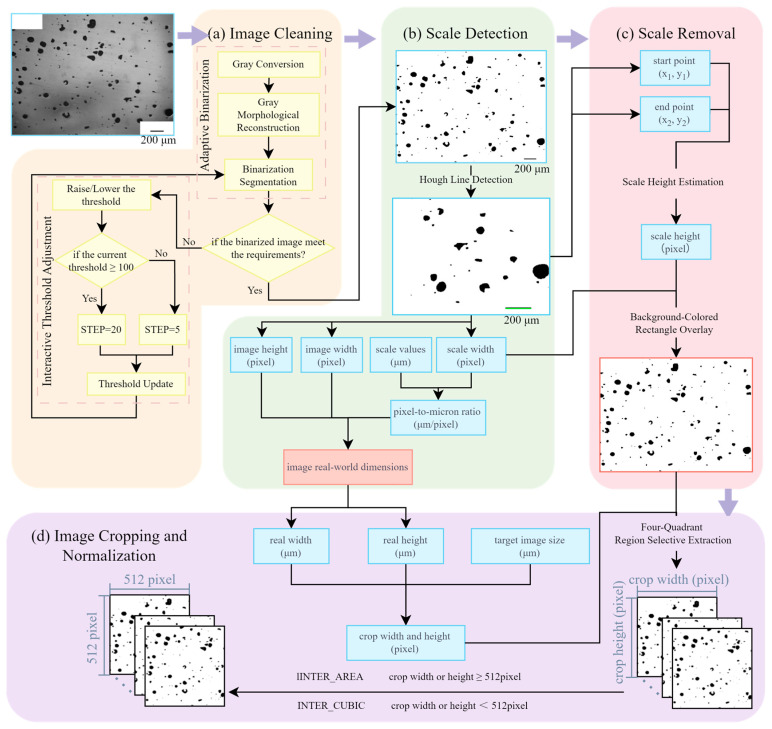
Schematic diagram of the proposed SLM multi-source porosity image integration method: (**a**) Image cleaning, (**b**) Scale detection, (**c**) Scale removal, and (**d**) Image cropping and normalization.

Adaptive binarization, a fundamental technique in image processing and computer vision, computes locally adaptive threshold values based on the pixel intensity distribution of the input image, enabling robust threshold-based segmentation. This method addresses the challenge of inconsistent background conditions across multi-source images, which often impedes their direct use in machine learning tasks. By converting images with varying backgrounds into a consistent binarized format, where white represents the background and black denotes pores—adaptive binarization facilitates standardized input for subsequent analysis. Consequently, this technique plays a crucial role in unifying the background and pore color representations across heterogeneous image datasets. The key processing workflow of adaptive binarization is illustrated in Figure 4a. For further implementation details of the adaptive binarization approach adopted in this study, please refer to Li et al. [20]. In addition, to further enhance binarization performance, this study incorporates dynamic threshold adjustment, which allows users to interactively fine-tune the threshold values based on visual inspection of the initial binary outputs, as exemplified in Figure 4a.

### 2.2. Scale Detection and Removal

The detection and removal of scale bars constitutes an important step in the standardization of multi-source microstructural images. In both experimental and literature-derived images, scale bars are typically introduced to convey physical dimension information and serve as the basis for converting pixel values into actual physical units. Establishing this conversion ratio is essential for harmonizing image dimensions across heterogeneous sources, enabling consistent cropping and resampling operations. However, scale bars are artificially introduced elements that do not represent intrinsic microstructural features of the material. Once the pixel-to-physical scale ratio has been determined, the presence of the scale bar becomes redundant and potentially detrimental. If left unprocessed, it may introduce noise and interfere with subsequent image analysis, especially in tasks such as pore segmentation, morphological feature extraction, and DL-based modeling. These artificial structures may be mistakenly identified as part of the actual microstructure, reducing analytical accuracy. Therefore, precise scale bar detection facilitates accurate physical unit calibration, while subsequent removal ensures structural integrity and consistency of the images, laying a reliable foundation for quantitative analysis and machine learning applications.

In this study, the physical scale of each microstructural image is determined by extracting the pixel length of the embedded scale bar present in the image. This approach enables precise conversion between pixel units and real-world physical dimensions, which is crucial for achieving comparability across multi-source datasets. Although DL-based object detection methods such as YOLO have shown impressive performance in general detection tasks, they typically require extensive annotated training data and GPU resources for effective deployment [29,30]. In contrast, our method is training-free, lightweight, and readily executable in local environments without specialized hardware or dependency on large-scale datasets. This makes it more practical for scenarios where scalable, high-throughput processing of diverse image sources is required. Moreover, it facilitates broader applicability and integration into customized image normalization pipelines without additional model tuning.

The detection process is primarily conducted in the lower half of the image, as observed from a survey of referenced literature-derived images, where scale bars are predominantly located in the lower region [31,32,33,34,35,36,37,38,39,40,41,42,43,44]. First, the image is converted to the HSV color space to facilitate color-based segmentation. Thresholds for red and black hues are then applied to generate masks that isolate potential scale bar regions. Specifically, for black scale bars, we define the HSV range as lower = (0, 0, 0) and upper = (180, 255, 30) to ensure robust detection against varying lighting conditions.

Contour-based detection is employed to locate candidate regions. To accurately isolate the scale bar from noise and microstructural features, only contours with a minimum area of 150 pixels^2^ and an aspect ratio between 5:1 and 30:1 are considered. Within the valid candidate regions, Canny edge detection is used to extract edge maps. Subsequently, the probabilistic Hough transform (Hough Lines [45]) is applied to detect straight line segments from the edge-detected image. Each edge point (*x*, *y*) is mapped into a parameter space using the transformation, as defined in Equation (1).

(1) ρ=xcosθ+ysinθ,

where ρ is the perpendicular distance from the line to the origin, and θ is the orientation angle. For the parameters of the probabilistic Hough transform, the distance resolution is set to *ρ* = 1 pixel and the angle resolution to *θ* = π/180 radians. The complete set of required parameters, including the threshold, minimum line length, and maximum line gap, is explicitly defined within Algorithm 1. Subsequently, a CnOCR-based text recognition [46] step is introduced to automatically extract the physical length value from the detected region. If the CnOCR detection fails, manual input of the physical length is requested as a fallback. Among all detected line segments, the longest horizontal line is selected as the scale bar candidate. The pixel length of this line, denoted as *L*_px_, is recorded. If the actual physical length of the scale bar is *L*_phys_, then the pixel-to-physical conversion ratio is given by Equation (2):

(2) $$Scale\;ratio=\frac{L_{\mathrm{phys}}}{L_{\mathrm{px}}}$$

Based on Equation (3), any measured pixel length *l*_px_ can be quantitatively converted to its corresponding physical length *l*_phys_, ensuring consistency across multi-source images.

(3) $$l_{\mathrm{phys}}=l_{\mathrm{px}}\times \frac{L_{\mathrm{phys}}}{L_{\mathrm{px}}}$$

After the scale information has been extracted, the scale bar must be removed to prevent it from interfering with structural segmentation. The removal process assumes that the detected line represents the scale bar and overwrites it using a white rectangular region or thick line. Let the coordinates of the detected line be (*x*_1_, *y*_1_) and (*x*_2_, *y*_2_), and let the estimated vertical thickness (height) of the scale bar in pixels be *h*. Then, as described in Equation (4), the region bounded horizontally by the detected scale bar endpoints (*x*_1_, *x*_2_) and vertically by the interval $\left[y_{1}-\frac{h}{2},y_{2}+\frac{h}{2}\right]$, is overwritten with white pixels to remove the scale bar from the image.

(4) $$Erase\;Region=\left[x_{1},x_{2}\right]\times \left[y_{1}-\frac{h}{2},y_{2}+\frac{h}{2}\right]$$

This operation ensures that the binary image used for analysis contains only valid microstructural features. The proposed scale bar detection and removal algorithms are detailed in Algorithms 1 and 2:
**Algorithm 1.** Automated Scale Bar Detection**Input**: grayscale or RGB image *P* after preprocessing**Output**: scale ratio *R* = *L*_phys_/*L*_px_, real-world dimensions of image *W*_real_, *H*_real_1:Image ← convert *P* to RGB if grayscale;2:$(H_{\mathrm{px}}$$,\;W_{px}$) ← Image.shape;3:$\mathrm{ROI}\;\leftarrow \;\mathrm{Image}[H_{\mathrm{px}}$//2:, :];//Define ROI as the lower half of *P*4:HSV ← Convert ROI to HSV color space;5:mask ← inRange(hsv, lower = (0, 0, 0), upper = (180, 255, 30));//Create black mask using HSV thresholds6:contours ← findContours(mask);//Extract contours from the mask7:Initialize *L*_px_ ← 0;8:**for** each contour **do**9: edge_img ← Canny(drawFilledContour(mask.shape, contour), 50, 150);//Apply Canny edge detection //Use probabilistic Hough transform to detect lines10: lines ← HoughLinesP(edge_img, rho = 1, theta = π/180, threshold = 50,        minLineLength = 50, maxLineGap = 10);11: **for** each detected line (*x*_1_, *y*_1_), (*x*_2_, *y*_2_) **do**12:  $\mathrm{Compute}\;\mathrm{length}\;l=\sqrt{{\left(x_{2}-x_{1}\right)}^{2}+{\left(y_{2}-y_{1}\right)}^{2}}$;13:  $\mathrm{if}\;l>L_{\mathrm{px}}$ then14:   *L*_px_ ← *l*;//Update *L*_px_15:*L*_phys_ ← CnOCR_detect_numeric_value(ROI);16:**if** *L*_phys_ = None then17: *L*_phys_ ← ask_user_input(“Please enter the actual length of scale bar (µm):”);//Prompt user to input physical length *L*_phys_18:$R\;\leftarrow \;L_{\mathrm{phys}}/L_{\mathrm{px}}$;//Compute scale ratio *R*19:$W_{\mathrm{real}}\;\leftarrow \;W_{\mathrm{px}}\cdot R$;//Compute real-world width of image20:$H_{\mathrm{real}}\;\leftarrow \;H_{\mathrm{px}}\cdot R$;//Compute real-world height of image21:**Return** *R*, *W*_real_, *H*_real_;
**Algorithm 2.** Scale Bar Removal**Input**: binary image *PB*, coordinates of scale bar endpoints (*x*_1_, *y*_1_), (*x*_2_, *y*_2_), estimated height *h* of the scale bar in pixels**Output**: image *PB* with scale bar removed1: ROI ← copy of *PB*;//Define ROI as the lower-right quarter of *P*2: HSV ← convert ROI to HSV color space;3: mask ← inRange(hsv, lower = (0, 0, 0), upper = (180, 255, 30));//Create black mask using HSV thresholds4: contours ← findContours(mask);//Extract contours from the mask5: Initialize *L*_px_ ← 0; //Step 1: scale bar detection6: **for** each contour **do**7: edge_img ← Canny(drawFilledContour(mask.shape, contour), 50, 150);//Apply Canny edge detection8: lines ← HoughLinesP(edge_img, rho = 1, theta = π/180, threshold = 50,        minLineLength = 50, maxLineGap = 10)9: **for** each detected line **do**10:  (*x*_1_, *y*_1_, *x*_2_, *y*_2_) ← line;11:  Compute length $l=\sqrt{{\left(x_{2}-x_{1}\right)}^{2}+{\left(y_{2}-y_{1}\right)}^{2}}$;12:  **if** $l>L_{\mathrm{px}}$ then13:   *L*_px_ ← *l*;//Update *L*_px_14:   *LT* ← int(*h*) + 1;//Define the height of scale bar //Step 2: scale bar removal15: drawLine(ROI, (*x*_1_, *y*_1_), (*x*_2_, *y*_2_), color = (255, 255, 255), thickness = *LT*)16: *PB* ← ROI;17: **Return** *PB*;


### 2.3. Image Cropping and Normalization

In multi-source porosity image integration, standardizing spatial scale is crucial for ensuring the comparability of heterogeneous data in downstream machine learning tasks. However, image datasets acquired from different studies often exhibit inconsistencies in physical area, resolution, and aspect ratio, which impair the structural interpretability and statistical reliability of pore features. Conventional resizing techniques, based solely on pixel dimensions, fail to preserve physical scale uniformity and may distort pore morphology. Therefore, an accurate and scalable cropping strategy that unifies the real-world dimensions of porosity images is essential for robust model training.

To address this issue, we introduce the final and most important step of the proposed Normalization for Multi-source SLM Porosity Images (NMI) framework: image cropping and resolution normalization, as illustrated in Figure 4d. This step ensures that all processed images share a consistent physical representation and pixel resolution, enabling fair comparisons and integration across datasets.

The foundation of this approach lies in translating the target physical dimensions into pixel-based cropping regions using image-specific scaling factors. First, the real-world dimensions of each image, including its physical width and height, are obtained using the pixel-to-micron ratio derived from scale bar detection, as outlined in Section 2.2. Simultaneously, the binarized pore image, free from annotation and scale bar interference, is retrieved from Section 2.1 for subsequent processing.

Next, a user-defined target image size (e.g., 700 µm × 700 µm) is used to determine the cropping region in physical units. This size is converted into pixel units by multiplying it by the inverse of the image’s pixel-to-micron scale factor, as defined in Equation (5). This ensures that each cropped region corresponds to the same physical area regardless of the source image’s original resolution.

(5) $$Crop\;Dimension_{\mathrm{pixel}}=Target\;image\;size_{\mu m}\times \left(\frac{1}{Scale\;ratio}\right)$$

To implement this, we apply a four-quadrant region selective extraction strategy, in which the image is divided into quadrants and cropped to match the specified physical area. This operation transforms all images into a uniform set with identical physical coverage while still preserving their native resolution [47].

Finally, to make the dataset directly compatible with machine learning models, we perform resolution standardization. For images with a resolution lower than 512 × 512 pixels, we use INTER_CUBIC interpolation (OpenCV2) for upsampling. For those exceeding the target resolution, INTER_AREA interpolation is used for downsampling. This guarantees that all images in the final dataset are of uniform resolution (512 × 512 pixels), ready for batch training or inference.

By explicitly aligning both the physical scale and pixel resolution, this step of the NMI framework effectively bridges the gap between heterogeneous porosity image sources. It preserves important structural features while enabling high-throughput, model-ready data generation, laying the groundwork for scalable and reliable SLM defect prediction using DL.

### 2.4. Interactive User Interfaces

In implementing the proposed framework, our work is oriented toward two primary goals. First, the standardization of heterogeneous porosity images can be conducted in a high-throughput, fully automated manner with minimal user intervention. In this context, image preprocessing consists of adaptive binarization, automatic scale bar detection, physical scale factor calculation (pixel-to-micrometer conversion), scale bar removal, and physical dimension-based cropping. These steps are executed as a batch process using either default or user-defined parameters. The cropped images are then rescaled to a unified resolution (e.g., 512 × 512 pixels) corresponding to a consistent physical target size defined by the user. This high-throughput processing enables seamless normalization of large-scale image datasets originating from diverse sources, such as experimental results and published literature.

**Figure 5:**
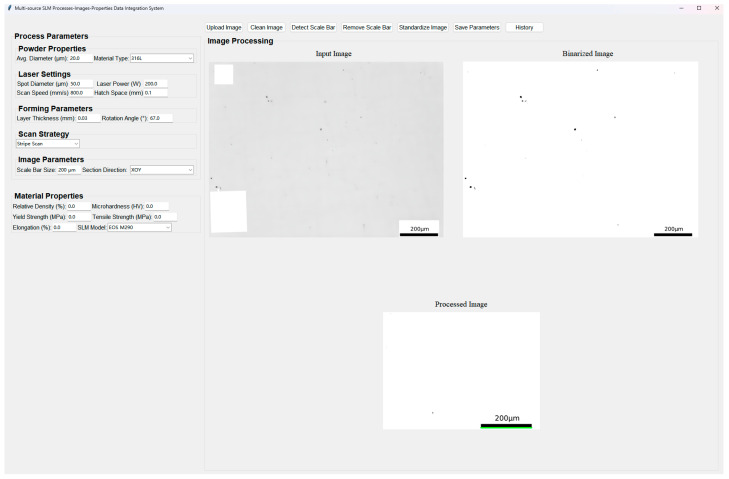
The proposed user-friendly interface for multi-source SLM data integration and visualization, built with Python 3.9 and OpenCV 4.9.

Second, a user-friendly data integration and visualization platform has been developed, as illustrated in Figure 5. This GUI-based interactive framework allows users to engage with each stage of the image standardization workflow. For instance, users can manually inspect and adjust binarization thresholds, verify scale bar detection results, or redefine cropping dimensions based on target physical size. The GUI also provides real-time visual feedback on preprocessing outcomes and statistical summaries of image characteristics (e.g., porosity distribution, resolution normalization status). Through the platform, users can perform manual interventions including: (a) refining binarization results, (b) visualizing intermediate steps such as pixel-to-physical scaling, and (c) exporting processed image sets for subsequent porosity analysis or model training. The platform was implemented using Python with PyQt for the interface and OpenCV for image operations.

An additional feature of this platform is the collection and structured storage of processes–properties data. Users can input or extract mechanical performance values (e.g., tensile strength, elongation, hardness) associated with specific process parameter sets (e.g., laser power, scan speed, hatch spacing), which are then saved as standardized .csv files for downstream analysis or dataset construction.

Using the developed platform, we processed and standardized a total of 111 multi-source images, completing the entire workflow in approximately 28 min. This corresponds to an average processing time of roughly 15 s per image, demonstrating the high-throughput capability of the framework for handling heterogeneous image datasets.

### 2.5. Error Propagation and Sensitivity Analysis

To comprehensively evaluate the impact of scale bar detection errors on the NMI pipeline, we established a systematic framework for error propagation analysis. This framework, validated through sensitivity analysis, quantifies how detection errors propagate through the pipeline and their influence on the physical size of cropped images.

As shown in Figure 4, the proposed NMI pipeline consists of several key steps: scale bar pixel length detection, scale bar text recognition, scale ratio computation, and uniform physical size of cropped images. In this process, let the detected scale bar pixel length be denoted as *L*_detected_, the extracted text value of the scale bar as $L_{\mu m}$, and the true scale bar pixel length as *L*_ture_. The relative detection error is defined as

(6) $$\varepsilon_{\det}=\frac{L_{\mathrm{detected}}-L_{\mathrm{true}}}{L_{\mathrm{true}}}$$

The scale ratio $R_{\mathrm{detected}}=L_{\mu m}/L_{\mathrm{detected}}$ (Equation (2)) is then used to compute the pixel length of the physical cropping region. To obtain the target physical size *S*_target_, the pixel region *C*_px_ is given by

(7) $$C_{\mathrm{px}}=S_{\mathrm{target}}/R_{\mathrm{detected}}$$

However, when using the true scale ratio *R*_true_ to calculate the actual physical size, we obtain

(8) $$S_{\mathrm{actual}}=C_{\mathrm{px}}\times R_{\mathrm{true}}=S_{\mathrm{target}}\times (1+\varepsilon_{\det})$$

Therefore, the relative error in physical size is shown as Equation (9).

(9) $$\varepsilon_{\mathrm{physical}}=\frac{S_{\mathrm{actual}}-S_{\mathrm{target}}}{S_{\mathrm{target}}}=\varepsilon_{\det}$$

This demonstrates that in NMI’s pipeline, the physical size error is directly proportional to the detection error, showing a 1:1 linear relationship with no error amplification. In addition, the sensitivity of the relative error in physical size is independent of the scale bar size. To validate the above theoretical model and quantify the system’s performance under different detection qualities, we conducted a systematic sensitivity analysis.

## 3. Data Preparation and Evaluation Metrics

### 3.1. Dataset Construction

According to ISO/ASTM TS 52930:2021 [48], the key process parameters that influence part quality include laser power, layer thickness, scanning speed, hatch spacing, spot size, and scanning strategy. To comprehensively assess the effects of process parameters on SLM-produced porosity images and corresponding mechanical properties, we collected a broad range of parameters from literature sources. These include laser power, scan speed, hatch spacing, layer thickness, spot diameter, scanning strategy, and interlayer rotation angle. Additionally, due to variations in experimental platforms across studies, equipment model, powder material type, and mean powder particle size were also recorded to account for device- and material-dependent influences. For performance data collection, this study focused on porosity-related metrics and mechanical performance under given process conditions, including porosity, ultimate tensile strength (UTS), yield strength, elongation at break, and microhardness.

Based on these criteria, a total of 473 process parameter sets related to SLM-fabricated 316L stainless steel were compiled from 30 peer-reviewed publications [31,32,33,34,35,36,37,38,39,40,41,42,43,44]. This includes 111 processes–porosity images groups and 400 processes-mechanical properties groups. Within the processes–images dataset, 63 groups were collected from literature sources, while 48 groups were obtained from a proprietary experimental dataset provided by Song et al. [31]. All data were either extracted from openly published articles in compliance with fair use policies or obtained directly with permission from the original authors, ensuring full adherence to copyright and data acquisition guidelines.

In this study, we define the following:

- The set of 63 literature-sourced porosity image groups as the literature-derived image dataset.
- The 48 experimental image groups as the experimental image dataset.
- Their union as the combined multi-source image dataset, totaling 111 porosity image groups. This third dataset uniquely integrates images obtained from both peer-reviewed publications and controlled experimental sources, thereby representing a heterogeneous fusion of multi-source data. It captures a broader range of process conditions, imaging modalities, and resolution scales, making it especially suitable for evaluating the robustness and generalizability of the proposed normalization framework.

**Figure 6:**
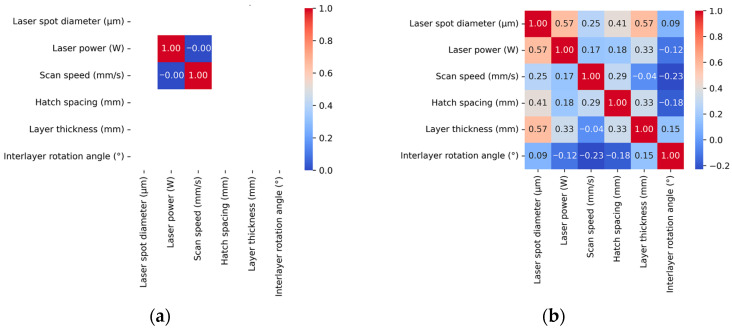
Correlation matrix heatmaps of key SLM process parameters: (**a**) Experimental dataset. (**b**) Literature-derived dataset, which demonstrates a broader coverage of parameter distributions compared to the experimental dataset.

Figure 6 illustrates the correlation matrix heatmaps of key parameters in both the literature-derived and experimental datasets. As shown in the figure, the literature-derived dataset exhibits broader parameter coverage in terms of laser spot diameter, hatch spacing, layer thickness, and interlayer rotation angle, reflecting the diversity of experimental setups reported across different studies. However, in the dimensions of laser power and scan speed, the experimental dataset demonstrates a wider exploration range. This is primarily because the experimental dataset was specifically designed to investigate the effects of these two parameters on porosity formation in SLM-fabricated specimens. Consequently, it includes extreme values such as exceptionally low scan speeds (100 mm/s) and exceptionally high laser powers (400 W), which are beyond the typical process parameter range and therefore rarely reported in the literature.

These findings highlight the complementary strengths of the two datasets: the literature-derived dataset ensures broad coverage of conventional and diverse process parameters, while the experimental dataset introduces critical edge cases, such as extreme scan speeds and laser powers, which are particularly valuable for porosity modeling. The integration of these datasets yields a more comprehensive and balanced foundation for robust data-driven analysis. To support the implementation of the proposed NMI method, all computations were performed on a system configured with Python 3.9, OpenCV 4.9, CUDA 12.4, and PyTorch 2.5, running on hardware equipped with a 13th Gen Intel^®^ Core™ i9-13950HX processor (Inter Corporation, Santa Clara, CA, USA).

### 3.2. Evaluation Metrics

To quantitatively evaluate the effectiveness of the proposed normalization framework (NMI), three evaluation metrics were used: the coefficient of variation (CV), the relative error of image porosity, and the error in the two-point correlation function (TPCF). These metrics were specifically selected to address the two core scientific questions posed in this study. The CV was used to assess the variation in physical area across cropped images, thereby directly reflecting the degree of spatial scale uniformity achieved by NMI and verifying its ability to ensure dimensional consistency across multi-source datasets. The relative error of image porosity was adopted to evaluate whether the porosity characteristics were preserved during the standardization process. Furthermore, the TPCF error was introduced to quantify discrepancies in spatial correlation of pore structures between normalized and original images. Since the TPCF characterizes the probability of finding two points belonging to the pore phase at a given separation distance, its error provides a sensitive measure of structural fidelity beyond global image porosity. By integrating these three metrics, a comprehensive assessment of NMI’s capability to eliminate multi-source porosity image data heterogeneity while maintaining critical structural information can be achieved.

1. Coefficient of Variation (CV). The CV is a standard statistical measure used to quantify relative dispersion within a dataset [49]. In this study, it is used to assess the consistency of physical image areas after normalization. It is calculated as Equation (10).
  (10) $$CV=\frac{\sigma }{\mu }\times 100\%,$$
  where σ is the standard deviation of image areas and μ is the mean. Lower CV values indicate better spatial uniformity across images.
2. Relative Error of Image Porosity. Image porosity is defined as the proportion of black pixels in a binarized microstructural image [31]. The relative error is computed by comparing the porosity of the original image with the average porosity of its cropped sub-images, as defined in Equation (11).
  (11) $$Relative\;Error_{\mathrm{Porosity}}=\frac{\left|{\bar{P}}_{\mathrm{sub}}-P_{\mathrm{orig}}\right|}{P_{\mathrm{orig}}}\times 100\%,$$
  where ${\bar{P}}_{\mathrm{sub}}$ denotes the average porosity across sub-images and $P_{\mathrm{orig}}$ represents the porosity of the original image. This metric evaluates whether the cropping and resolution standardization process preserves critical structural features related to porosity.
3. TPCF Error. The TPCF error was calculated as the mean absolute error between the normalized and original TPCF curves [31], as expressed in Equation (12).
  (12) $$E_{\mathrm{TPCF}}=\frac{1}{N}\sum_{i=1}^{N}\left|S_{2}^{\mathrm{norm}}(r_{i})-S_{2}^{\mathrm{orig}}(r_{i})\right|,$$
  where $S_{2}^{\mathrm{norm}}(r_{i})$ and $S_{2}^{\mathrm{orig}}(r_{i})$ denotes the values of the two-point correlation function at distance r for the normalized and original images, respectively, and *N* is the total number of sampled distances. Lower $E_{\mathrm{TPCF}}$ values indicate higher structural fidelity of normalized images.
4. Roundness. The roundness is a geometric measure used to quantify the closeness of a pore’s shape to a perfect circle. It is calculated using the ratio of the area *A* to the square of the perimeter *P* of the pore [50], as shown in Equation (13).
  (13) $$Roundness=\frac{4\pi A}{P^{2}},$$
  where *A* is the area of the pore, and *P* denotes the perimeter of the pore. A roundness value closer to 1 indicates a shape more similar to a perfect circle, while lower values indicate more irregular or non-circular shapes.
5. Average pore area. The average pore area is a measure used to quantify the mean size of pores in an image. It is calculated by averaging the area of all detected pores within the image [51], as expressed in Equation (14).
  (14) $$Average\;Pore\;Area=\frac{1}{n}\sum_{i=1}^{n}A_{i},$$
  where *n* is the total number of pores in the image, and *A_i_* denotes the area of the *i*-th pore. In this study, the average pore area is used to assess the overall size distribution of pores across images.

## 4. Results and Discussions

### 4.1. Evaluation of Automatic Scale Extraction Accuracy

To ensure that the automatically extracted scale bars accurately reflect the physical dimensions in the images, we evaluated the performance of the proposed scale detection method using 63 image samples collected from the literature. The pixel length of the scale bar in each image was manually measured at the pixel level and used as ground truth for quantitative evaluation. We further analyzed the relative error between the scale lengths obtained by the proposed morphology-based automatic measurement method and the ground truth. For comparison, the results obtained through manual annotation using ImageJ software and measurements derived from the LIST image analysis platform developed by Kim et al., and the widely used YOLOv8 model for object detection were also included. In order to adapt YOLOv8 for scale bar detection, a YOLOv8 model was trained for 500 epochs on a publicly available scale bar detection dataset sourced from Roboflow (https://universe.roboflow.com/scanning-electron-microscopy/scalebar-va3kp, accessed on 14 November 2025). The trained model was subsequently applied to the 63 image samples for scale bar detection.

**Figure 7:**
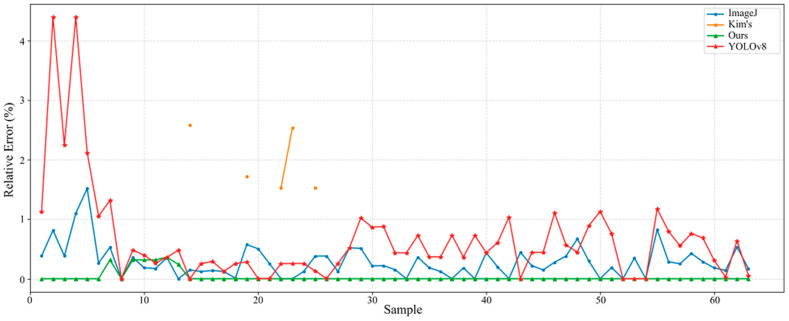
Comparison of the relative error in scale bar pixel length between the manually annotated ground truth and the results obtained using ImageJ, the LIST image analysis platform, YOLOv8, and the proposed automatic scale bar detection method.

Figure 7 presents a comparative analysis of the relative error in scale bar detection across 63 literature-derived image samples, evaluating the proposed automatic scale bar detection method against manual annotation using ImageJ, the LIST platform, and the YOLOv8 model. As shown in Figure 7, the proposed method achieves highly accurate and reliable detection results, comparable to those of manual annotation, thereby confirming its validity and robustness. The evidence for this includes: (i) Among the 63 image samples, the maximum relative error between the scale bar pixel length measured by the proposed automatic method and the ground truth remained below 0.4%, with 90% of the measurements showing zero relative error. (ii) In 95% of the image samples, the relative error of the scale bar pixel length obtained using the automatic method was lower than that of the manual annotation method. Compared with the LIST image analysis platform, the proposed method also demonstrates significantly higher detection success rates and measurement accuracy: it successfully detected scale bars in all 63 samples, whereas the LIST platform succeeded in only 5 samples. Moreover, the LIST-derived results exhibited relative errors exceeding 1.5%, which is higher than those achieved by our approach. This advantage is particularly important in the context of literature-derived datasets, where image quality, format, and labeling conventions vary widely. By achieving full detection success and sub-percent error levels across all samples, the proposed method establishes a solid basis for subsequent quantitative analyses, such as pore size normalization and cross-study data harmonization. The enhanced detection stability also minimizes human subjectivity and potential measurement bias inherent in manual annotation, thereby improving data reliability and reproducibility.

Additionally, to further assess the performance of modern deep learning detectors for scale bar measurement tasks, the YOLOv8 model was compared with the proposed method. YOLOv8 was able to successfully detect the scale bar region, text information, and corresponding pixel lengths across all 63 image samples, showing good generalization across varying backgrounds, contrasts, and formats. However, as shown in Figure 7, the measurement accuracy of YOLOv8 was notably lower than that of both manual annotations and the proposed automatic detection method. Specifically, although YOLOv8 achieved a 100% detection success rate, its relative error ranged from 0.6% to 2.8%, significantly higher than the proposed method, which maintained an error level below 0.4%; In over 85% of the test images, YOLOv8 exhibited a higher relative error than the proposed method, and in 78% of the samples, its error was also higher than that of ImageJ manual annotations. The primary source of error in YOLOv8 stemmed from the bounding box regression mechanism inherent in object detection: the model outputs a rectangular box that must enclose both the scale bar entity and its text, and even small shifts in the boundary position can lead to significant errors in the measurement of scale bar pixel lengths, especially in cases of short scale bars or thin lines.

In contrast, the proposed method, based on contour extraction and sub-pixel level positioning, avoids the systematic bias introduced by bounding box prediction, resulting in superior detection accuracy and stability. Additionally, YOLOv8 requires a large amount of annotated data for training, whereas the proposed method is fully automatic, requiring no training, and is better suited for handling literature images of varying resolutions, noise characteristics, and formats. These results demonstrate that, although YOLOv8 shows strong robustness in scale bar detection, it still cannot match the proposed method in terms of measurement accuracy and repeatability, further validating the advantages of the proposed method in large-scale image quantification and cross-study data standardization.

Table 1 demonstrates that the proposed automatic scale bar detection method significantly reduces measurement time while maintaining high accuracy. Further analysis indicates that the time required for three researchers to perform scale bar detection using the automatic method was only 38.81% of the time required when using ImageJ for manual measurement. A paired t-test conducted on 63 images revealed a statistically significant difference between the two methods (*p* < 0.01), confirming the efficiency gain. This suggests that the proposed automatic scale bar detection method not only meets the accuracy requirements for scale detection across multi-source images but also significantly reduces manual effort, achieving a 61.19% improvement in efficiency. These results highlight the practical advantage of the proposed method in large-scale image data preprocessing, particularly when dealing with heterogeneous datasets from multiple literature and experimental sources.

Moreover, the detection results from YOLOv8 further validate the effectiveness of the proposed method. Despite YOLOv8 demonstrating certain advantages in automated detection, its overall time consumption remains relatively high, primarily due to the inclusion of model training time. In comparison to the automatic method, which only involves the detection process, YOLOv8’s time consumption is significantly higher, as repeatedly confirmed in several experiments. Therefore, when considering the training time, YOLOv8’s detection efficiency still cannot match that of the proposed method. In terms of accuracy, YOLOv8’s error rate is slightly higher than that of the proposed method. Specifically, YOLOv8’s average relative error is approximately three times that of manual annotations, while the proposed method closely matches the manual measurement results, with errors remaining minimal, almost never exceeding 10% of the manual method’s error. This indicates that the automatic scale bar detection method not only ensures high accuracy but also greatly improves efficiency, particularly when processing large-scale image datasets.

Based on the data analysis presented above, the proposed automatic scale bar detection method saves considerable manual time and computational resources while ensuring high-quality and consistent data. Particularly in the preprocessing of large-scale image data, automation reduces the need for human intervention and minimizes the bias and errors inherent in manual detection. This advantage makes the proposed method highly practical and scalable, especially in building multi-source heterogeneous datasets for supporting machine learning and deep learning applications. This advantage is especially crucial in literature image datasets, where image quality, formats, and annotation methods exhibit high variability. By achieving 100% successful detection and sub-percent error levels across all samples, the proposed method lays a solid foundation for subsequent quantitative analysis.

**Figure 8:**
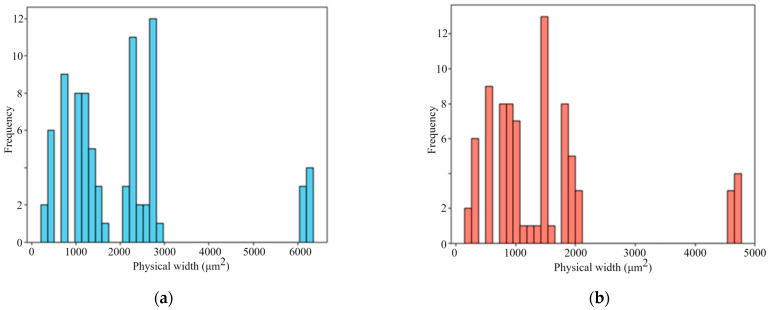
Frequency distribution of physical dimensions from Literature-Derived Images: (**a**) Frequency distribution of physical widths. (**b**) Frequency distribution of physical heights.

Figure 8 illustrates the physical size distribution of the literature-derived images, calculated using the scale factors extracted by the proposed automatic scale bar detection algorithm. It can be observed that the physical dimensions of the images within the dataset are highly inconsistent, with substantial variation across samples. This finding highlights the necessity of unifying physical image dimensions when integrating multi-source data. Therefore, the crop size is carefully chosen to ensure compatibility across datasets of different scales. A fixed crop size of 700 µm × 700 µm is adopted based on a quantitative constraint: ensuring that at least 90% of the collected images can be fully cropped without any resizing. This crop size also takes into account the typical pore size range of SLM 316L stainless steel (generally 10–100 µm) [52], ensuring that a 700 µm field of view contains a sufficient number of pores and their spatial distributions for statistically reliable feature extraction.

**Figure 9:**
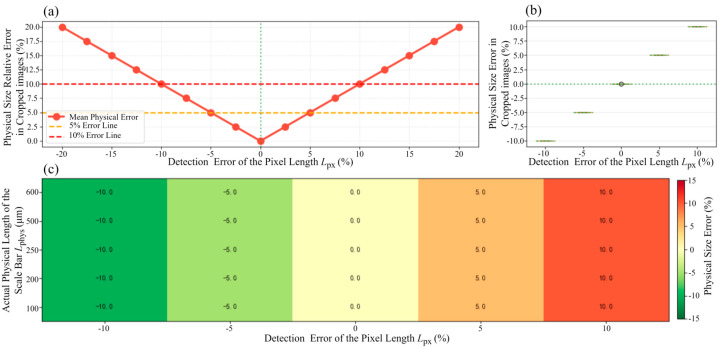
Quantification of Error Propagation and Sensitivity Analysis for Scale Bar Detection Errors of Cropped Images: (**a**) Error Propagation of Physical Size Relative Error in Cropped Images for Scale Bar Detection Error. (**b**) Sensitivity of Physical Size Error to Detection Error in Cropped Images. (**c**) Impact of Actual Physical Length of the Scale Bar and Detection Error of the Pixel Length on Physical Size Error.

Furthermore, to validate the influence of scale bar detection accuracy on the subsequent processing steps of the NMI pipeline, we conducted a corresponding sensitivity analysis. We uniformly sampled 17 points within the detection error range of [−20%, +20%]. For each error level, we calculated the physical size errors for all 111 test samples. Figure 9 presents the quantification of error propagation and sensitivity analysis for scale bar detection errors in cropped images.

Figure 9a,b together show the relationship between detection error and the final physical size error in cropped images. As predicted by the theoretical model, these two exhibit a near-perfect linear relationship, with a slope close to 1.0, confirming the 1:1 nature of error propagation. Across the 111 test samples, the coefficient of determination (R^2^) for this linear relationship was found to be greater than 0.999, indicating a high degree of consistency between the model’s predictions and experimental observations. As the scale bar detection error increases, the physical size error increases linearly; for example, a ±10% detection error results in an approximate ±10% final size error.

The sensitivity analysis results presented in Figure 9b show that the distribution, median, and variance of the final physical size error remain consistent and centered around 0%, despite variations in experimental parameters such as image resolution, the presence of scale bar text, and cropping area sizes. This strongly suggests that the physical size error is primarily determined by the accuracy of scale bar pixel length detection, with negligible influence from other image characteristics or cropping setup factors. Finally, Figure 9c presents a heatmap showing the effects of the scale bar’s actual physical length and the pixel length detection error. This analysis reveals that, regardless of the scale bar’s actual physical length, the calculated relative physical size error is almost entirely determined by the relative error in pixel length detection. This finding demonstrates that the critical factor requiring stringent control during the dimensional conversion process is the accuracy of scale bar pixel detection.

### 4.2. Comparison of Multi-Source Data Before and After NMI

Accurate scale detection enables precise estimation of image physical dimensions, which is essential for ensuring consistency in physical size across multi-source images processed by the NMI method. To evaluate the effectiveness of the proposed NMI method in addressing scale inconsistencies across multi-source images, we applied NMI to three datasets: (1) an experimental image dataset, (2) a literature-derived dataset, and (3) a combined multi-source image dataset, using a fixed crop size of 700 µm × 700 µm as the target image size. As a baseline for comparison, we adopted the single-source image processing workflow proposed by Song et al. [31], enabling a direct assessment of NMI’s performance against a conventional single-source cropping approach.

Table 2 shows the physical area distributions resulting from two kinds of image processing methods, as well as no processing, applied to three types of datasets: those acquired from experimental measurements, collected from the literature, and a combined multi-source dataset. As shown in the table, interestingly, NMI-processed images exhibited significantly higher consistency in physical area compared with those processed using the single-source cropping method or left unprocessed. This is supported by the CV values of the physical areas in all three datasets processed using the NMI method, being as low as 0.06%, while the CV values of the other two image processing approaches both exceeded 100% in the literature-derived dataset and the combined multi-source dataset. This significant improvement arises because literature-sourced images inherently possess dimensional heterogeneity due to variations in magnification and resolution across studies, making it challenging to align scales directly (as shown in Figure 6). By incorporating automatic scale detection and normalization, the NMI method effectively standardizes the physical dimensions across datasets, outperforming single-source cropping in cross-scale consistency. Moreover, the NMI method achieves precise physical area control by cropping images of various actual dimensions to a unified target physical size. Specifically, the mean deviation between each image’s actual physical area and the target crop area is 0%, 0%, and 0.04% for the three datasets, with the latter two datasets showing a remarkably low standard deviation of only 0.0003 mm^2^. These results clearly demonstrate that the NMI method effectively eliminates dimensional inconsistencies and enables reliable integration of heterogeneous multi-source images into a unified dataset suitable for quantitative analysis and machine learning applications.

**Figure 10:**
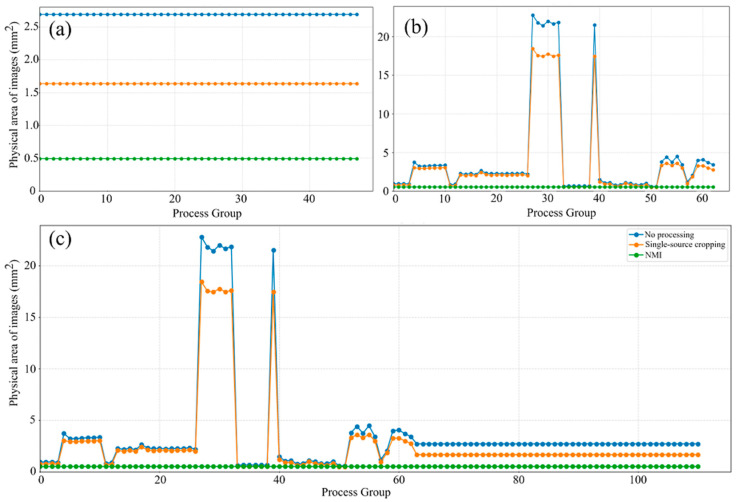
Distribution of image physical areas across three SLM porosity datasets processed using different normalization methods: (**a**) Experimental dataset. (**b**) Literature-derived dataset. (**c**) Combined multi-source dataset. Each subfigure illustrates the physical area of images under three processing conditions: unprocessed original images, single-source cropping, and the proposed NMI method, highlighting the normalization effect across varying data sources.

Figure 10 illustrates the distribution of image physical areas across three datasets processed with different normalization methods. In the experimental dataset shown in Figure 10a, the physical area remains nearly constant under all three methods, reflecting the uniform acquisition conditions and limited variability. In contrast, the literature-derived dataset presented in Figure 10b exhibits pronounced differences in the unprocessed state due to variations in resolution, magnification, and cropping across studies. Although the single-source cropping method reduces some of these discrepancies, inconsistencies among processing groups persist. For example, the physical areas of groups 27 to 32 and group 39 exceed 20 mm^2^, whereas most other groups are below 5 mm^2^. After applying the NMI method, all groups are rescaled to a consistent physical dimension and aligned along the same horizontal level in the distribution plot, effectively eliminating cross-group inconsistencies and achieving standardized spatial representation. For the combined multi-source dataset (Figure 10c), the discrepancies between unprocessed and cropped images are even more pronounced, underscoring the challenges of directly integrating heterogeneous datasets. Once again, the NMI method demonstrates strong normalization capability, unifying all image groups to a consistent physical scale and thereby ensuring reliable analysis of porosity features.

Another interesting observation from Figure 10c is that the NMI method not only normalizes the physical dimensions of images collected from the literature, but also adjusts the experimental images to the same target size. The evidence for this is that the original physical area of the images obtained from experiments was consistently 2.6906 mm^2^, which was reduced to 0.49 mm^2^ after applying the NMI method, indicating successful alignment with the standardized target size. This implies that the NMI method enables the seamless integration of private experimental datasets, thereby expanding the overall dataset and enhancing its utility for subsequent analysis. In contrast, the single-source cropping method is less effective than the NMI method in addressing the issue of multi-scale image normalization. Despite some variations in scale, the single-source cropping method exhibited an overall similar trend for the physical area curves in Figure 10b,c, closely following the pattern of the original images but with reduced magnitudes along the vertical axis. This is because the single-source cropping method operates based on image resolution without considering the actual physical area represented by that resolution, and hence, the NMI method is able to compensate for this limitation by standardizing the physical dimensions across images.

### 4.3. Impact of Scale Standardization on Porosity Feature Distribution

To provide a quantitative evaluation of the normalization quality for multi-source SLM pore images, this case study adopts the relative error of image porosity, as introduced in Section 3. Both metrics are preferable when yielding lower values, indicating higher consistency and fidelity after normalization.

**Figure 11:**
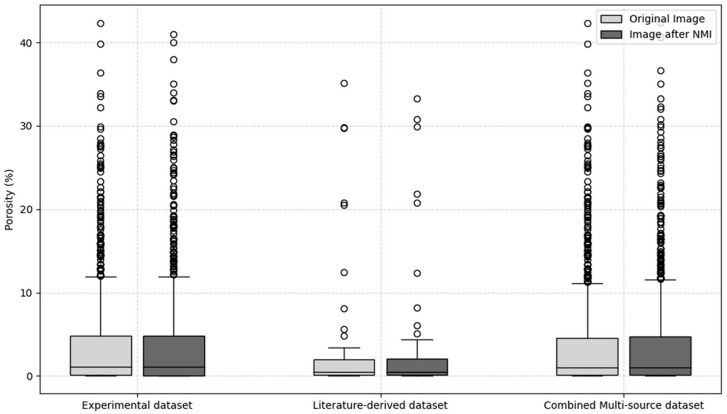
Box plots of image porosity distribution based on NMI-processed and original images applied to the experimental dataset, literature-derived dataset, and the combined multi-source dataset. Small circles represent outliers beyond 1.5 times the interquartile range (IQR) from the box edges.

Figure 11 compares the image porosity of the original unprocessed images with the average image porosity of the image groups generated by the NMI method, covering the experimental dataset, the literature-derived dataset, and the combined multi-source dataset. In the experimental dataset, the image porosity of the original images is nearly identical to that of the NMI-processed groups. The distributions remain stable in terms of median, interquartile range (IQR), and overall range, indicating that in the set of images obtained through experiments, the NMI method introduces no distortion and preserves the reliability of quantitative porosity measurements. For the literature-derived dataset, where substantial variability arises from differences in magnification, resolution, and cropping strategies, the comparison is more illustrative. Even under heterogeneous conditions, the original and NMI-processed images exhibit a high degree of consistency: the medians are nearly identical, and the IQR of the NMI-processed images is 1.8%, which is close to the value of 1.9% observed in the original images. A small number of outliers are observed in both cases, but these can be attributed to inherent differences in the source studies rather than to the normalization procedure itself. These results confirm that NMI preserves the statistical distribution of image porosity while unifying spatial scales. Furthermore, the results from the combined multi-source dataset in Figure 11 highlight the feasibility of integrating image data collected from the literature with those obtained through experiments. Consistent with the findings from the previous datasets, the NMI-processed image groups exhibit stable image porosity characteristics, with the median essentially unchanged, the IQR remaining comparable, and the overdistribution shape well preserved. These results indicate that NMI effectively eliminates inconsistencies caused by scale differences while maintaining both central tendency and variability. By unifying image scales without altering image porosity measurements, NMI provides a robust foundation for cross-dataset comparison and integration, ensuring accuracy and objectivity in quantitative microstructural analysis. This capability is particularly critical for consolidating large-scale heterogeneous image collections to support statistical investigations or machine learning modeling.

**Figure 12:**
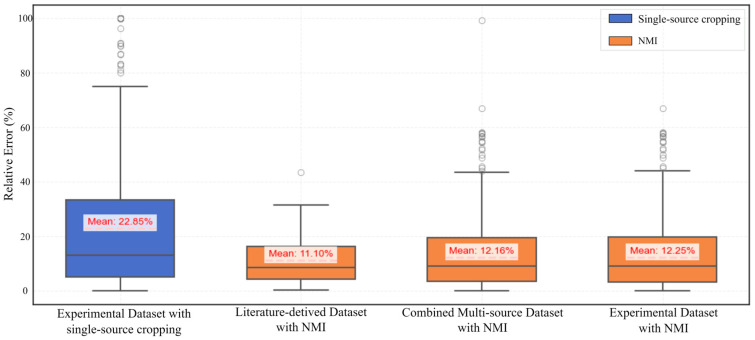
Box plots of the relative error of image porosity based on single-source cropped experimental images and NMI-processed images from the experimental dataset, literature-derived dataset, and the combined multi-source dataset. The comparison highlights the effectiveness of the NMI method in reducing porosity error and improving consistency.

Figure 12 provides a comparative overview of the relative error in image porosity across different datasets, based on single-source cropped experimental images and NMI-processed images. The box plots illustrate the distribution of error values for the experimental dataset, the literature-derived dataset, and the combined multi-source dataset.

Across the dataset obtained from the experiment, the NMI-processed images exhibit notably lower median values (9.0%) and reduced variability in relative error, as reflected by a narrower interquartile range (IQR: 16.5%), compared to the single-source cropped counterparts, which show a higher median (13.1%) and a much wider IQR (28.4%). This indicates improved stability and consistency in porosity quantification. This conclusion is supported by the statistical metrics derived from the experimental dataset. Specifically, the NMI-processed images have a median relative error of 9.0% and an interquartile range (IQR) of 16.5%, compared to 13.1% and 28.4%, respectively, for the single-source cropped images. Moreover, the whisker range for NMI-processed images spans from 21.5% to 44.5%, while the single-source cropping method yields a much broader range, from 37.7% to 76.0%. Additionally, the number of outliers in the NMI group (18) is significantly lower than that in the single-source cropped group (31). These quantitative indicators collectively demonstrate that NMI processing leads to reduced dispersion and fewer extreme deviations, thus providing a more stable and reliable basis for porosity quantification. These findings demonstrate the robustness of the NMI method in harmonizing spatial scales while preserving porosity characteristics, enabling reliable and consistent quantification across heterogeneous sources.

It can also be observed that, across the experimental, literature-derived, and combined multi-source datasets, the NMI-processed images consistently yield lower median relative error values and narrower interquartile ranges (IQRs). In contrast, the single-source cropped experimental images exhibit larger error ranges and multiple outliers, particularly in the combined dataset, suggesting greater sensitivity to resolution differences and dataset heterogeneity. This conclusion is further corroborated by statistical results across all datasets. In the literature-derived dataset, the NMI-processed images yield a low median relative error of 8.6% with a narrow IQR of 12.0%, and only one outlier is detected, indicating minimal variability. In the combined dataset, the NMI-processed images show a median of 9.0%, an IQR of 16.1%, and 19 outliers, significantly fewer than in the experimental single-source cropped group, which contains 31 outliers and exhibits a broader IQR of 28.4%. These trends consistently demonstrate that the NMI method not only reduces relative error magnitude but also suppresses variability and extreme deviations across heterogeneous datasets. The comparative stability achieved by NMI highlights its robustness in handling differences in resolution and source heterogeneity, particularly in multi-source integration scenarios.

### 4.4. Comparison of NMI and Single-Source Cropping in Maintaining Pore Spatial Correlation

In Section 4.3, we compared the relative porosity errors obtained using the NMI method and the single-source cropping approach across three datasets. The results demonstrated that, in terms of statistical characteristics, the NMI method not only achieves scale normalization of multi-source porosity images without altering their intrinsic porosity values, but also enhances the robustness of the datasets by mitigating extreme deviations. To further evaluate the capability of the proposed NMI method in handling heterogeneous multi-source datasets, this section investigates its impact on the spatial correlation of pore structures. The correlation was quantitatively assessed using the TPCF error introduced in Section 3, with the single-source cropping results (averaged over 50 trials) serving as the reference baseline.

**Figure 13:**
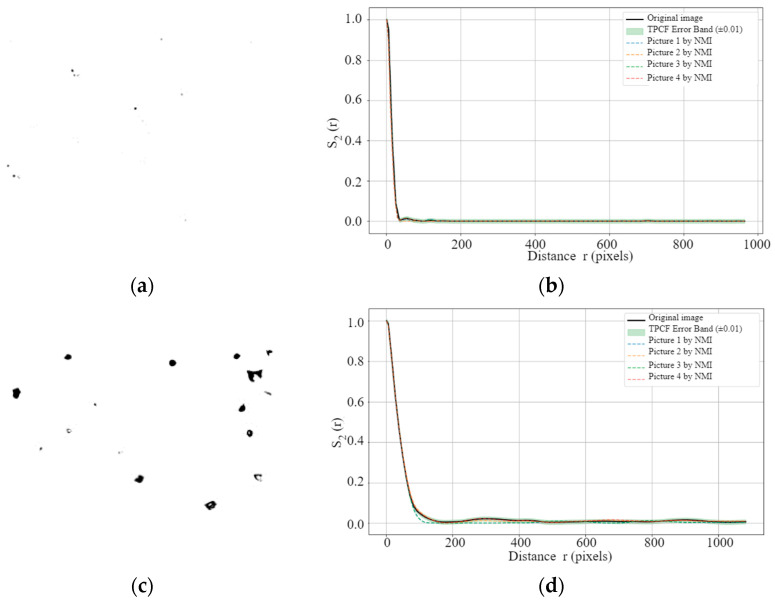

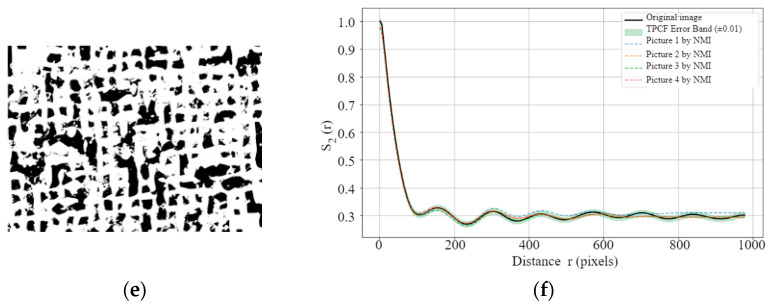
TPCF curves of original and NMI-processed images with three porosity levels. (**a**,**c**,**e**) show the original images with porosities of 0.08%, 0.89%, and 29.75%, respectively. (**b**,**d**,**f**) present the corresponding TPCF curves, where the black line denotes the original image and the colored lines represent the TPCF curves of image groups processed by the NMI method.

Table 3 summarizes the average, maximum, and median TPCF errors ($E_{\mathrm{TPCF}}$) obtained from single-source cropping applied to images with different porosity levels. From these results, it is evident that porosity has a significant influence on the discrepancy between cropped images and their original counterparts. Specifically, as porosity increases from 0.08% to 29.75%, the average $E_{\mathrm{TPCF}}$ rises by more than fourfold, while the maximum $E_{\mathrm{TPCF}}$ exhibits an increase of at least 500%. Furthermore, Table 3 suggests that, compared with the average and median $E_{\mathrm{TPCF}}$, the maximum $E_{\mathrm{TPCF}}$ provides a more reliable criterion for defining acceptable discrepancies after image processing. Although the average TPCF error remains within ±0.01 for all cases, the maximum $E_{\mathrm{TPCF}}$ exceeds this threshold by 36.46% for the 0.89% porosity sample B (Figure 13c) and by 70.65% for the 29.75% porosity sample C (Figure 13e). These findings indicate that setting the permissible TPCF error of NMI-processed images relative to the original images based on the maximum error obtained from single-source cropping is a rational and conservative approach.

Building on this benchmark, we further examined the TPCF curves of NMI-processed images at different porosity levels to directly evaluate their ability to preserve spatial correlations of pore structures. Figure 13 presents the TPCF curves of the original and NMI-processed images at porosity levels of 0.08%, 0.89%, and 29.75%. In Figure 13b, Figure 13d and Figure 13f, the green shaded regions represent the acceptable error bands relative to the original images, defined as ±0.003, ±0.013, and ±0.017, respectively. The results show that, for all three kinds of images, the discrepancies between the NMI-processed and original images largely remain within these ranges. In some cases, the deviations are even smaller than the more stringent thresholds given by the average and median $E_{\mathrm{TPCF}}$. This indicates that the normalization process preserves the spatial distribution characteristics of pore structures [53,54] and that the processed images are sufficient for training ML models [11].

Furthermore, Figure 13d,f reveal an important trend: as porosity increases, localized deviations beyond the error bands may occur, even though the overall TPCF errors remain below the defined thresholds. This can be attributed to the difference between global error metrics and localized curve fluctuations. While global metrics such as average and median $E_{\mathrm{TPCF}}$ capture the overall consistency between curves, local deviations highlight the scale sensitivity of pore structure distributions. In high-porosity images, the larger pore sizes and their tendency to cluster increase sensitivity at certain spatial scales, leading to temporary departures from the acceptable bands despite overall errors remaining within tolerable limits. Consequently, these findings confirm that the NMI method effectively standardizes multi-source porosity images while preserving spatial correlation structures, with robustness across different porosity levels.

**Figure 14:**
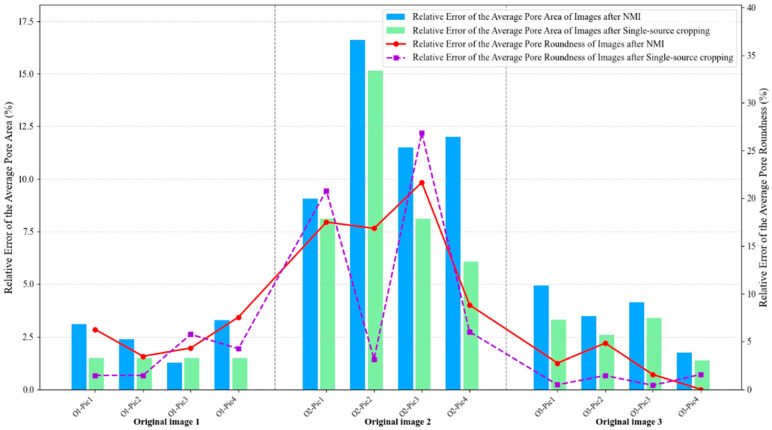
Relative error (compared to original images) in average pore area (bar chart, left *Y*-axis) and roundness (line plots, right *Y*-axis) for NMI-processed and single-source cropped images with three porosity levels. For comparison, each original image is processed by two methods (NMI-processed and single-source cropping) to obtain four representative normalized images. The *X*-axis labels (e.g., “O1-Pic1”) denote the specific processed image, where ‘O1’ refers to Original Image 1 (representing Porosity Level 1) and ‘Pic1’ is the first representative picture derived from that original image after processing.

From Figure 14, it can be clearly observed that NMI and single-source cropping methods exhibit comparable performance for both average pore area and roundness errors in original images 1 and 3, representing the extreme porosity levels. For original image 1 (0.08% porosity), both methods maintain low errors with pore area errors ranging from 1% to 3% and roundness errors between 1% and 6%, showing closely matched trajectories across all four representative images. Similarly, for original image 3 (29.75% porosity), both methods achieve even lower errors with area errors at 2% to 5% and roundness errors at 0% to 5%, with the bar heights and line trajectories remaining nearly identical. These consistent results at both porosity extremes indicate that when processing images with either simple morphology (few pores with clear boundaries) or high statistical stability (hundreds of pores providing averaging effects), the NMI method has minimal impact on morphological feature preservation. Interestingly, it is observed that original image 2 (0.89% porosity) exhibits substantially elevated errors for both methods and both metrics, yet NMI demonstrates superior consistency, particularly for roundness preservation. For pore area errors (bar charts), both methods show comparable maximum values, with NMI ranging from 9% to 17% and single-source cropping spanning 8% to 15%. However, the critical distinction emerges in roundness errors (line plots), where NMI exhibits a relatively smooth trajectory between 8% and 21%, while single-source cropping demonstrates erratic fluctuations from 3% to an alarming 27%, with dramatic jumps between adjacent cropped regions (from 17% at O2-Pic2 to 27% at O2-Pic3, then plummeting to 8% at O2-Pic4). This stark contrast reveals that although neither method can fully eliminate errors when processing challenging medium-porosity images, NMI provides significantly more predictable and stable results across different cropping positions, especially for shape-related metrics.

In addition, it is also observed from Figure 14 that roundness errors consistently exceed area errors across all three images for both methods, with this disparity being most pronounced in Image 2. For Images 1 and 3, the gap between the two metrics remains modest (approximately 2 to 4 percentage points), but for Image 2, roundness errors substantially surpass area errors by factors of 1.2 to 1.8, with the purple dashed line peaking at 27% while the corresponding green bar reaches only 15%. This systematic pattern can be attributed to the computational nature of roundness, which depends on both area and perimeter measurements, making it inherently more sensitive to boundary distortions introduced during resampling and interpolation. Small pixel-level changes in pore boundaries, which produce only minor area calculation variations (reflected in the relatively stable bar charts at 9% to 17%), can disproportionately alter perimeter measurements and consequently amplify roundness errors (manifested in the more volatile line plots reaching 21% to 27%). Furthermore, the elevated errors specific to Image 2 can be quantitatively explained by its unique position in the resolution loss spectrum: despite possessing the highest original resolution (2.74 pixels/μm), Image 2 experiences the most aggressive 3.75-fold downsampling from 1918 × 1918 to 512 × 512 pixels, causing medium-sized pores (10 to 30 μm diameter) that originally spanned 27 to 82 pixels to be compressed into merely 7 to 22 pixels. This severe compression destroys boundary details while the moderate pore population (10 to 20 pores) proves insufficient for statistical averaging, unlike Image 3, where hundreds of pores effectively dilute individual distortions despite comparable 3.12-fold resolution loss, or Image 1, where minimal 2.85-fold downsampling preserves adequate detail for the few pores present.

Through the evaluations presented above, including the assessment of automatic scale extraction accuracy, statistical comparisons of physical area and porosity distributions, TPCF analyses of pore spatial correlations, and quantitative assessments of morphological feature preservation, the robustness and effectiveness of the proposed NMI method are comprehensively demonstrated. Compared with both the conventional single-source cropping approach and unprocessed images, NMI exhibits superior performance in several critical aspects. It not only ensures dimensional and resolution consistency across heterogeneous datasets but also preserves intrinsic pore morphology and spatial correlation characteristics, as evidenced by the stable porosity distributions and low TPCF errors. These results indicate that NMI effectively eliminates cross-source discrepancies without introducing distortions, thereby producing a unified and high-fidelity dataset. Such consistency is particularly beneficial for DL applications, where model performance strongly depends on data homogeneity and structural integrity. The NMI-processed datasets enable DL models to extract pore-related morphological features more efficiently and to learn more reliable process–structure–property relationships. Consequently, this normalization framework establishes a solid foundation for developing accurate and generalizable DL models for porosity detection, defect prediction, and mechanical property estimation in SLM and other metal additive manufacturing processes.

### 4.5. Performance Evaluation of the NMI Method in Image Generative and Porosity Predictive Modeling

To quantify the performance improvement of machine learning models using NMI normalized data and to evaluate the application of multi-source processes–properties datasets in training porosity prediction models, we conducted two primary experiments. The first experiment involved training two image generation models using normalized and non-normalized data, respectively, to assess the effect of data normalization on model performance. The second experiment employed the processes–porosities dataset to train seven supervised machine learning algorithms, including Support Vector Machine (SVR), Decision Tree Regressor (DTR), Random Forest Regressor (RFR), Gradient Boosting Regressor (GBR), Gaussian Process (GPs), K-Nearest Neighbors (KNN), and Multi-layer Perceptron (MLP) [52]. These models were evaluated based on the coefficient of determination (R^2^), the mean squared error (MSE), and the root mean square error (RMSE).

For the pore image generation model, this study selected the Denoising Diffusion Probabilistic Model (DDPM) [55], an image generative model widely applied for fine structure generation. This model learns data distributions through a multi-step iterative denoising process and possesses advantages including rich image detail generation, excellent modal diversity, and high training stability. These characteristics make DDPM particularly suitable for generating fine structures such as microscopic pores that are strongly correlated with process parameters. The training dataset contained pore images of different sizes and scales. The input consisted of 256 × 256 pixel single-channel pore images and corresponding SLM process parameters, including laser power, scanning speed, layer thickness, hatch spacing, scanning strategy, average powder particle size, and laser spot diameter. Process parameters were encoded into 128-dimensional feature vectors through a 3-layer fully connected network and subsequently fused with image features extracted by the U-Net backbone network through concatenation at the second encoder layer. The network structure comprised 6 downsampling modules and 6 upsampling modules, with SiLU activation functions and Adam optimizer (learning rate 1 × 10^−4^, β_1_ = 0.9, β_2_ = 0.999). The diffusion process was set to 1000 steps, ultimately generating pore images corresponding to specified process parameters.

**Figure 15:**
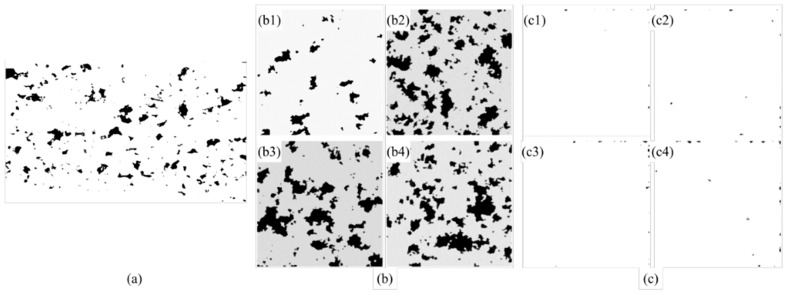
Comparison of generated images of DDPM trained with non-normalized and NMI-normalized data: (**a**) Real binarized pore image corresponding to the process parameters. (**b**) Generated image from the DDPM trained with normalized data, with four representative images labeled as (**b1**–**b4**). (**c**) Generated images from the DDPM trained with non-normalized data, with four representative images labeled as (**c1**–**c4**).

From Figure 15, it can be clearly observed that the DDPM trained with NMI-normalized data demonstrates substantial improvements in pore morphology consistency and scale uniformity compared to the model trained with non-normalized data. As shown in Figure 15b, the generated images from the normalized model exhibit well-defined pore structures with consistent morphological features across all four representative samples (Figure 15(b1–b4)). The pores display uniform size distribution and regular geometric shapes that closely resemble the real binarized pore image shown in Figure 15a. In contrast, the images generated by the non-normalized model (Figure 15(c1–c4)) exhibit significant morphological distortions and inconsistent pore characteristics, demonstrating poor correlation with the target structure in Figure 15a.

Interestingly, it is observed that the normalized model maintains remarkable spatial homogeneity in pore distribution throughout the generated images. The density and spatial arrangement of pores in Figure 15(b1–b4) remain consistent with each other and with the reference image, indicating that the model has successfully learned the underlying relationship between process parameters and pore formation patterns. The pores exhibit natural boundaries and realistic shapes that are characteristic of actual SLM-manufactured samples. This generation capability suggests that NMI effectively resolves the scale inconsistency issues present in the multi-source dataset, enabling the model to establish accurate mappings between process parameters and corresponding microstructural features.

In the second experiment, we trained seven supervised machine learning algorithms using the collected processes–porosities dataset to predict porosity values based on corresponding process parameters. This experiment aimed to evaluate the effectiveness of multi-source processes–properties datasets in materials performance prediction using machine learning techniques, with particular emphasis on porosity prediction. The study was benchmarked against the research conducted by Barrionuevo et al. [56] and Alamri et al. [57], who similarly performed performance predictions based on porosity and mechanical property data collected from multiple literature sources.

First, our collected processes–porosities dataset comprised 332 data groups, each associated with specific process parameters. In comparison to Barrionuevo et al. [56], who focused solely on laser power, scanning speed, layer thickness, hatch spacing, and particle size, and Alamri et al. [57], who considered only laser power, scanning speed, layer thickness, and hatch spacing, we recognized the additional influence of laser spot size, scanning strategy, and inter-layer rotation angle on the performance of SLM manufactured parts according to the ISO/ASTM TS 52930:2021 [48] standard. Therefore, to mitigate the impact of varying printing equipment and other confounding factors inherent in multi-source processes–properties datasets collected from different literature sources, we comprehensively considered all process factors relevant to SLM printing quality.

Second, to evaluate the validity of our comprehensive approach to process parameter selection in multi-source processes–properties datasets, we adopted the seven supervised machine learning regressors (SVR, DTR, RFR, GBR, GPs, KNN, and MLP) proposed by Barrionuevo et al. [56] for predicting the relative density of 316L stainless steel samples produced by SLM processes. We compared the training results on Barrionuevo et al.’s [56] original dataset (Baseline) with the model prediction results on our extended multi-source processes–properties dataset that incorporated additional process parameter categories (Extended), with the comparative results presented in Table 4.

From Table 4, it can be clearly observed that the Extended dataset consistently outperforms the Baseline dataset across all seven ML algorithms. All models showed R^2^ improvements ranging from 3.4% to 102.8%, with corresponding MSE reductions of 42.2% to 81.0%. Interestingly, it is observed that the GPs exhibited the most remarkable improvement, achieving a 102.8% R^2^ increase (from 0.4039 to 0.8192) and an 81.0% MSE reduction, transforming from the poorest to the secondly best performer. In addition, it is also observed from Table 4 that even high-performing baseline algorithms demonstrated significant gains. The RFR improved by 23.7% in R^2^ with a 35.9% RMSE reduction, while the GBR achieved a 23.3% RMSE decrease. This consistent enhancement pattern validates that incorporating additional process parameters (laser spot size, scanning strategy, and inter-layer rotation angle) universally improves model predictive capability. It can be conclusively deduced that the inclusion of three additional process parameters (laser spot size, scanning strategy, and inter-layer rotation angle) in the Extended dataset fundamentally enhances the machine learning models’ ability to capture the complex relationships between SLM process parameters and porosity formation, suggesting that comprehensive consideration of equipment specific and process control factors is essential for accurate porosity prediction in multi-source datasets.

## 5. Conclusions

In this study, a normalization method (NMI) is proposed for integrating multi-source porosity images in SLM, addressing the challenge that porosity images from different experiments and literature sources often exhibit heterogeneity in scale and resolution, which prevents their direct use in ML and DL model training. In this framework, we develop an automatic scale detection and removal procedure to extract scale information from multi-source images, convert it into a scale ratio, and eliminate the interference of the scale bar on pore structure features. Then, the scale ratios are applied in the image cropping and normalization stage, ensuring that all images are rescaled to the same physical region and resolution.

To validate the proposed methodology, three representative datasets are employed for evaluation: a literature-derived dataset, an experimental dataset, and a combined multi-source dataset. The results are compared against methods commonly adopted in previous studies. Based on both statistical features, such as CV and relative porosity error, as well as structural correlation features quantified by TPCF errors, the results consistently demonstrate that NMI effectively eliminates inconsistencies across multi-source datasets. It ensures dimensional consistency while preserving the spatial correlation of pore structures. These findings confirm that NMI substantially enhances the feasibility of integrating experimental and literature-derived data. This capability provides a new pathway for data acquisition to support DL predictive modeling of porosity defects and mechanical performance in SLM, thereby accelerating processes in additive manufacturing.

It is worth noting that the proposed method is specifically designed for optical porosity images of SLM-fabricated metallic materials, such as 316L stainless steel, where consistent illumination and texture patterns facilitate accurate normalization. Although the framework can, in principle, be extended to optical images of other alloys after suitable parameter tuning, its applicability to non-optical imaging modalities (e.g., XCT or SEM) remains limited due to fundamental differences in image contrast and formation mechanisms.

For future work, we plan to apply the normalized multi-source datasets processed by NMI to predict porosity and key mechanical properties, thereby further demonstrating the practical utility and generalization potential of the proposed method. Moreover, we aim to explore its adaptability to different imaging modalities by integrating contrast enhancement and domain adaptation techniques, enabling more universal image normalization and feature consistency across diverse data sources.

## Figures and Tables

**Table 1:** The overall error and time consumption were compared between the manually annotated ground truth and the results obtained using ImageJ, YOLOv8, and the proposed automatic scale bar detection method.

Method	Mean Absolute Error ^1^ (MAE)	Mean Relative Error ^2^ (MRE, %)	Time (min)
ImageJ	0.001884	0.1794%	42 ± 5
YOLOv8	0.005762	0.5957%	31.4
Automatic scale measurement (Ours)	0.000742	0.0714%	16.3 ± 1.5

^1^ Mean Absolute Error: the average of the absolute differences between the measured values and the reference values. ^2^ Mean Relative Error: the average of the absolute differences between the measured values and the reference values, normalized by the reference values.

**Table 2:** Comparison of physical area distribution statistics before and after normalization across three dataset types, using both the single-source cropping and the proposed NMI method.

Dataset Type	Process Groups	Image Processing Method	Mean Physical Area (mm^2^)	Standard Deviation (mm^2^)	Coefficient of Variation (CV, %)
Experimental	48	-	2.6906	0.0000	0.0000
		Single-source cropping	1.6339	0.0000	0.0000
		NMI	0.4900	0.0000	0.0000
Literature-derived	63	-	5.7838	8.3928	145.1077
		Single-source cropping	3.5085	5.0946	145.2065
		NMI	0.4900	0.0003	0.0612
Combined	111	-	3.5479	4.8101	135.576
		Single-source cropping	2.6979	3.9489	146.3691
		NMI	0.4898	0.0003	0.0601

**Table 3:** TPCF errors from single-source cropping applied to images with varying porosity levels.

Image Sample	Porosity (%)	Average *E*_TPCF_	Max *E*_TPCF_	Median *E*_TPCF_
A	0.08%	0.001839	0.003124	0.001718
B	0.89%	0.008332	0.013646	0.008846
C	29.75%	0.008759	0.017065	0.007640

**Table 4:** Comparative results from seven supervised ML regressors applied to SLM 316L porosity prediction with Baseline (5 parameters) and Extended (8 parameters) datasets.

ML Algorithm	R^2^		∆R^2^	MSE		∆MSE	RMSE		∆RMSE
	Baseline	Extended		Baseline	Extended		Baseline	Extended	
SVR	0.6033	0.6425	↑^1^6.5%	0.3807	0.2144	↑43.7%	0.6170	0.4631	↑24.9%
DTR	0.4248	0.6638	↑56.3%	0.6223	0.1963	↑68.5%	0.7888	0.4489	↑43.1%
RFR	0.6033	0.7462	↑23.7%	0.3709	0.1434	↑61.3%	0.6090	0.3903	↑35.9%
GBR	0.6296	0.6512	↑3.4%	0.3554	0.2091	↑42.2%	0.5961	0.4572	↑23.3%
GPs	0.4039	0.8192	↑102.8%	0.5719	0.1084	↑81.0%	0.7563	0.3296	↑56.4%
KNN	0.5288	0.8012	↑51.5%	0.4521	0.1192	↑73.6%	0.6724	0.3452	↑48.7%
MLP	0.5745	0.6678	↑16.2%	0.3979	0.1916	↑51.8%	0.6308	0.4464	↑29.2%

^1^ ↑: the percentage improvement in prediction metrics achieved by the Extended dataset relative the Baseline dataset.

## Data Availability

The original contributions presented in this study are included in the article. Further inquiries can be directed to the corresponding author.
